# Reduction in *Plasmodium falciparum Pfk13* and *pfg377* allele diversity through time in southern Vietnam

**DOI:** 10.1186/s41182-022-00409-4

**Published:** 2022-03-01

**Authors:** Nguyen Quang Thieu, Vu Duc Chinh, Truong Van Hanh, Nguyen Van Dung, Hidekazu Takagi, Takeshi Annoura, Satoru Kawai, Gaku Masuda, Nguyen Van Tuan, Vu Viet Hung, Shusuke Nakazawa, Richard Culleton, Nguyen Thi Huong Binh, Yoshimasa Maeno

**Affiliations:** 1 National Institute of Malariology, Parasitology and Entomology, Hanoi, Vietnam; 2 Department of Microbiology and Immunology, Aichi Medical University School of Medicine, Aichi, Japan; 3 Department of Parasitology, National Institute of Infectious Diseases, Tokyo, Japan; 4 Laboratory of Tropical Medicine and Parasitology, Dokkyo Medical University, Tochigi, Japan; 5 Department of International Affairs and Tropical Medicine, Tokyo Women’s Medical University, Tokyo, Japan; 6 School of Tropical Medicine and Global Health, Nagasaki University, 1-12-4 Sakamoto, Nagasaki, Nagasaki 852-8523 Japan; 7 Faculty of Medical Engineering, Phenikaa University, Hanoi, Vietnam; 8 Department of Protozoology, Institute of Tropical Medicine, Nagasaki University, Nagasaki, Japan; 9 Division of Molecular Parasitology, Proteo-Science Centre, Ehime University, Ehime, Japan; 10 Department of Virology and Parasitology, Fujita Health University School of Medicine, Aichi, Japan

**Keywords:** *Plasmodium falciparum*, Artemisinin resistance, K13-propeller gene, pfg377 gene, Gametocyte, Vietnam

## Abstract

**Background:**

*Plasmodium falciparum* has acquired resistance to artemisinin in Southeast Asia, with mutations in the *P. falciparum* Kelch-13 (*Pfk13*) gene associated with the resistance phenotype. The widespread use of Artemisinin-based combination therapy (ACT)s in Southeast Asia has led to the selection and spread of parasites carrying mutations in *Pfk13*. We characterised the allele diversity of *Pfk13* and *pfg377*, an artemisinin-resistance neutral polymorphic gene, in parasite DNA extracted human blood from in southern Vietnam in 2003, 2012, 2015 and 2018.

**Method:**

This study was conducted in Bu Gia Map commune, Binh Phuoc province, Vietnam, from May 2018 to January 2019. Twenty-four samples from 2018 to 2019, 30 from 2003, 24 from 2012 and 32 from 2015 were analysed. Malaria-infected human blood was collected by finger-prick and used for molecular analysis. A nested-PCR targeting the small subunit ribosomal RNA gene was used for *Plasmodium* species identification, followed by amplification and nucleotide sequencing of *Pfk13* and region 3 of *pfg377*. Archived blood samples collected in the same region in 2012 and 2015 were also analysed as above for comparison.

**Results:**

The genetic diversity of *Pfk13* and *pfg377* was lower in 2018–2019 compared to 2012 and 2015. The number of distinct *Pfk13* mutants decreased from three in 2012 and 2015, P553L, V568G and C580Y, to one, C580Y in 2018–2019. In 2018–2019, the frequency of C580Y mutant strains was 71% (17/24 isolates). All samples were wild type in 2003. In 2012 and 2015, there were single-strain infections as well as co-infections with two mutant strains or with mutant and wild strains, whereas there were no co-infections in 2018. *pfg377* allele diversity decreased from five alleles in 2012 to two alleles in 2018–2019.

**Conclusion:**

The genetic diversity of *P. falciparum* was reduced at the two genetic loci surveyed in this study, *Pfk13* and *pfg377*. In the case of the former gene, we observed an increase in the prevalence of parasites carrying the C580Y gene, known to confer reduced susceptibility to ACTs. The reduction in the diversity of *pfg377* may be linked to the clonal expansion of parasite strains carrying the C580Y mutation, leading to an overall reduction in parasite genetic diversity across the population.

**Supplementary Information:**

The online version contains supplementary material available at 10.1186/s41182-022-00409-4.

## Background

Artemisinin-based combination therapy (ACT) is currently the most common first-line treatment for *Plasmodium falciparum* in most malaria-endemic countries. However, in recent years, there have been various reports of artemisinin resistance in various countries in the Great Mekong subregion, such as Cambodia, Thailand, Myanmar, Laos, China, and Vietnam [1–6].

It appears that mutations in the propeller domain of the gene encoding the Kelch 13 (K13) protein (*Pfk13*), are associated with delayed parasite clearance in the presence of artemisinin in vitro and in vivo [7, 8]. In Southeast Asia, parasites carrying mutations in the *Pfk13* gene at positions C580Y, Y493H, R539T, and I543T have been observed, and these have been linked to artemisinin resistance phenotypes. The major mutations in Vietnam are C580Y, P574L, V568G, P553L, I543T, and Y493H, all of which were detected in southern Vietnam near the border with Cambodia [9]. There are reports that resistant malaria has spread from Cambodia, but the emergence of resistant *P. falciparum* has also been observed in other areas of Vietnam, where importation of parasites seems unlikely [10, 11]. There have been various reports on such mutant strains, but no reports on co-infections of mutant strains and their degree of artemisinin resistance.

Artemisinin and semisynthetic derivatives including artesunate, artemether and dihydroartemisinin, are short-acting antimalarial agents that kill parasites more rapidly than conventional antimalarials and are active against both the sexual and asexual stages of the parasite [12].

A range of polymorphic gametocyte-specific antigen-encoding genes have previously been described in *P. falciparum*, including *pfs230*, *pfs16*, *pfs25*, *pfg27/25* and *pfg377* [13, 14]. Region 3 of *pfg377* is particularly useful as a gametocyte marker, due to allelic heterogeneity in its molecular weight [15, 16]. Seven allele types have been reported for this gene, and four of these have been reported from asymptomatic *P. falciparum* carriers in Binh Phuoc Province, Vietnam [17].

In this report, we characterise polymorphisms in *Pfk13* and *pfg377* using parasite DNA extracted from blood samples collected from individuals resident in Southern Vietnam over the course of 8 months from May 2018 to January 2019. We describe changes in their allele prevalences through time with reference to archived samples collected from 30, 24, and 32 individuals in the same region in 2003, 2012, and 2015, respectively.

## Materials and methods

**Fig. 1:**
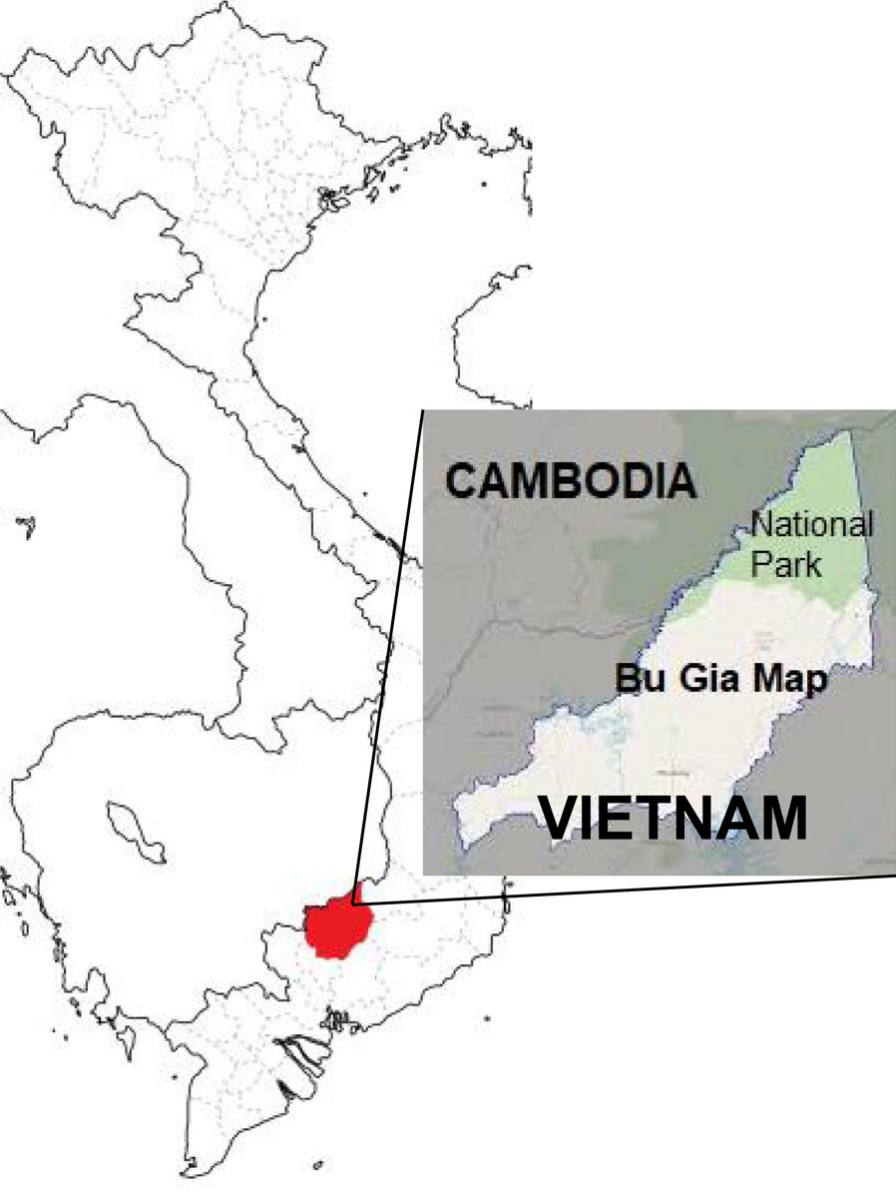
Geographical map of Bu Gia Map commune, Binh Phuoc Province as the study area

This study was conducted in Bu Gia Map commune (11° 56′ N; 106° 59′ E), Binh Phuoc province, Vietnam (Fig. 1), from May 2018 to January 2019. Forty-eight patients aged 3–61 years (mean ± standard deviation: 28 ± 12 years), who visited the commune health center with fever above 37.5 °C and were diagnosed as malaria by Rapid Diagnostic Test (RDT) (SD Malaria Ag P.f/P.v, Standard Diagnostics Inc., Kyonggi-do, Republic of Korea) were enrolled in the study. There were 47 male and 1 female patients. Body temperature ranged from 37.7 to 39.6 °C (mean ± standard deviation: 38.5 ± 0.5 °C) (Table 1a). To characterise changes in allele prevalence through time, we also examined sex- and age-matched samples from *P. falciparum* malaria patients collected and stored in 2003, 2012, and 2015. The number of samples for the comparison years 2003, 2012, and 2015 were 30, 24, and 32, respectively. Treatment was performed according to Vietnamese Ministry of Health guidelines. This study was reviewed and approved by the research ethics committee of the National Institute of Malariology, Parasitology, and Entomology, Ministry of Health, Vietnam (366/QD118-VSR) and by the ethics committee of Fujita Health University, Japan (HM17-050) and Nagasaki University, Japan (10121662-5 and NU_TMGH 2020_125_1). Patients were informed of the objectives, processes, and procedures of this study, and written or oral informed consent was obtained. Blood was collected by finger-prick; some of the collected blood was used for testing by RDT and the rest was applied to filter paper for molecular analyses. Each blood-spotted filter paper was immediately air dried, placed in a sealed plastic bag, and stored at room temperature.

**Table 1:** Characteristics of patients infected with *Plasmodium* parasites (a) in Bu Gia Map commune, Binh Phuoc Province, and diagnosis results of *Plasmodium* species in samples of human blood samples (b)

a. Characteristics of the study patients				
No. examined (*Plasmodium* positive cases)	48			
Age (years)	3 to 61 (mean ± SD: 28 ± 12)			
Sex	47 male, 1 female			
Body temperature (°C)	37.7 to 39.6 (mean ± SD: 38.5 ± 0.5)			
Estimated location of infection	Total	Pf	Pv	Pf + Pv
In the National Park	36	18	17	1
In the commune	12	5	7	0

b. Malaria diagnosis results of the study sample				
Infected *Plasmodium* species	Microscopy	RDT	PCR	
Pf	24	24	23	
Pv	24	24	24	
Pm	0	–	0	
Pf + Pv	0	0	1	

Pf: *P. falciparum*; Pv: *P. vivax*; Pm: *P. malariae*; Pf + Pv: mixed infection with Pf and Pv

Genomic DNA was extracted from dried blood spots stored on filter paper using the QIAamp DNA Micro Kit (Qiagen, Tokyo, Japan) as previously described [17, 18]. An 18S rRNA gene-based nested PCR was used for the detection and identification of *P. falciparum* and other *Plasmodium* species as previously described [11]. Amplification of the *Pfk13* gene and region 3 of the gametocyte-specific gene *pfg377* was carried out by nested PCR as previously described [11, 17], and the products or target DNA insertion plasmids were sequenced with BigDye Terminator v3.1 Cycle Sequencing Premix Kit (ABI, Foster city, CA, USA). Sequencing products were run on an ABI/Hitachi 3130 × 1 Genetic Analyzer (ABI) and nucleotide sequences were analysed using Genetyx (Genetyx Corporation, Tokyo, Japan). Double peaks at the same position in the electropherogram of the sequence of *Pfk13*, in which the secondary peak was greater than 50% of the amplitude of the primary peak, were considered mixed strain-infections (Additional file 1: Fig. S1).

## Results and discussion

Blood samples from patients in 2018–2019 were examined by PCR for the presence and species of *Plasmodium* parasites. The number of single and mixed *Plasmodium* species infections was 47 and 1, respectively. There was a total of 23 samples containing *P. falciparum*, 24 containing *P. vivax* and one with a mixed infection of *P. falciparum* and *P. vivax* (Table 1b). *Anopheles dirus* from this area have previously been shown to be infected with both human and non-human primate *Plasmodium* parasites [19], but no infection of humans with non-human primate *Plasmodium* parasites was observed in this study. Body temperatures of falciparum and vivax malaria patients averaged 38.6 °C and 38.5 °C, respectively, with no significant difference between them.

Patients were interviewed about the location of their infection acquisition at the time of their first medical examination, and it was found that for both falciparum and vivax malaria patients that infection in national parks was three times more common than in communes (Table 1). There was no difference in the location of infection acquisition of *P. falciparum* and *P. vivax*. That the risk of infection and transmission is higher in the national park area is supported by the results of a vector mosquito survey carried out in the same period [19].

**Fig. 2:**
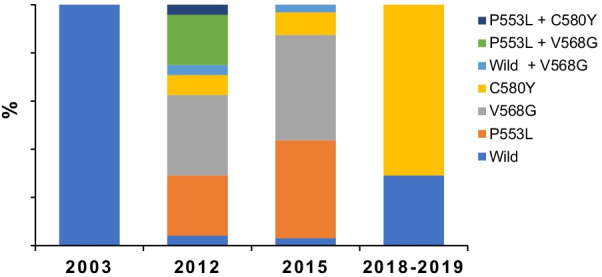
Prevalence of Pfk13 mutations in *Plasmodium falciparum* in Binh Phuoc province, 2003 to 2019

Among the 48 *Plasmodium* positive samples, 24 *P. falciparum* positive samples were analyzed to determine the nucleotide sequence of the nested PCR amplified portion of *Pfk13* (nucleotide positions 1279–2127). Single nucleotide polymorphisms with respect to the *Pfk13* sequence of the 3D7 clone (PF3D7_1343700) were recorded. Seventeen out of 24 (71%) *P. falciparum* infected samples in 2018–2019 presented mutations, with only non-synonymous mutations observed. The same analysis was carried out for the 2003, 2012 and 2015 samples. In 2003, all 30 samples analyzed were wild type; in the 2012 and 2015 samples, 31 of 32 (97%) and 23 of 24 (96%) were mutant strains, respectively (Fig. 2).

In 2018–2019, 17 samples with the C580Y were recorded with no other mutations found (Fig. 2). In both 2012 and 2015, single infections by strains carrying mutant alleles and co-infections of strains carrying mutant alleles were detected. In these years, three mutant alleles, P553L, V568G, and C580Y, were present. Three types of co-infections were recorded: wild type + V568G, P553L + V568G, and P553L + C580Y (Fig. 2). To determine whether these non-synonymous mutant alleles were unique to the study area, we selected and surveyed neighbouring and remote areas with similar environments to the study site and compared the results. The species and frequency of mutant alleles in this study area and neighbouring areas were similar, but remote areas showed different results with less frequent mutations [11, 20]. V568G and P553L were the dominant mutants in the 2012 and 2015 samples, whereas C580Y was detected at a low frequency in these years (Fig. 2). These results are consistent with another study carried out on this area [20]. As of 2016, 28 non-synonymous substitution mutant alleles of *Pfk13* have been described in Southeast Asia, 16 of which were reported from artemisinin-resistant samples. On the west side of the Mekong River, China, Myanmar and Thailand, F446I, N458Y, P674L and R561H are dominant, while on the east side, Cambodia, Laos and Vietnam, C580Y, R539T, Y493H and I543T are dominant. All these mutant alleles have been detected in resistant samples. Among these mutants, C580Y is dominant in Thailand and Cambodia, as in this study. Of the three variants detected in this study, P553L and V568G are the only mutants that were mainly detected in Vietnam. These are also the mutants that have been reported since the early detection of mutant strains [9, 21–23].

**Fig. 3:**
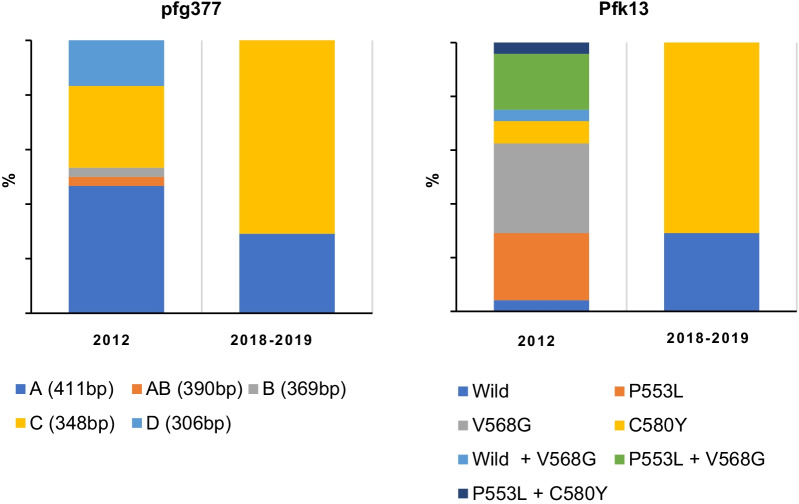
Comparison of the prevalence of *Pfk13* mutants and *pfg337* alleles between 2012 and 2018–2019

The relative proportions of the various mutations in *Pfk13* vary from year to year. In the presented study, there was no association between the number or diversity of *Pfk13* alleles and drug resistance or infection efficiency. To determine if this reduction in genetic diversity was observable at other genetic loci, we next examined the polymorphic gametocyte-specific gene *pfg377*. The number of alleles of *pfg377* in the study area ranged from five in 2012 to two in 2018–2019 (Fig. 3), mirroring the reduction in allele diversity observed for *Pfk13*, which changed from 7 in 2012 to 2 in 2018–2019 (Fig. 3). There was also no relationship between *Pfk13* mutation diversity and that of the *pfg377* allele.

The proportion of parasites with *Pfk13* mutation in this study was consistent with previous studies carried out in the same area [20]. Unlike previous studies, however, co-infection with multiple mutant strains were detected in the same patient samples. Consistent with this, five alleles of *pfg377* were found in 2012, when a mixed infection of three *Pfk13* mutants was also observed. This reduced to two types each for both *Pfk13* and *pfg377* in 2018–2019.

The C580Y mutation confers a moderate level of resistance against artemisinin and its derivatives. This mutation is the most widespread in the Mekong region, and it is replacing other mutations in areas, where other mutations were originally dominant [24].

The acquisition of mutations that confer drug resistance can occasionally result in fitness costs relative to wild types in the absence of drug pressure. The Pfk13 C580Y mutation results in less of a fitness cost than other mutations [25]. However, the fitness cost of C580Y alone cannot explain the rapid spread of this substitution [25]. Genome-wide association analysis suggests that mutations in genes other than *Pfk13* may further compensate for fitness.

The relative prevalence of *P. falciparum* at each time point studied in Binh Phuoc Province, where Bu Gia Map is located, has decreased (Additional file 1: Table S1). A reduction in the effective population size of the parasite pool would result in a decrease in the level of genome-wide diversity. This may be one of the factors contributing to the reduced in the diversity of mutant strains.

Nineteen of 24 cases of falciparum malaria were contracted in the Bu Gia Map national park. The study area, including this national park, is part of a closed border area with Cambodia, and local ethnic minorities have little interaction with other places, especially at night. Therefore, the invasion of new variants from exotic sources is unlikely, suggesting that the changes in allele prevalences observed in this region are the result of changing environmental conditions for the local parasite population, rather than the result of import of strains from outside.

## Conclusion

The genetic diversity of *P. falciparum* was reduced at the two genetic loci surveyed in this study, *Pfk13* and *pfg377*. In the case of the former gene, we observed an increase in the prevalence of parasites carrying the C580Y gene, known to confer reduced susceptibility to ACTs. The reduction in the diversity of *pfg377* may be linked to the clonal expansion of parasite strains carrying the C580Y mutation, leading to an overall reduction in parasite genetic diversity on the population.

The results of this epidemiological study suggest that the genetic polymorphisms created by ACTs pressure are eliminated due to fitness issues, and the spread of the remaining C580Y mutation is a future issue for malaria control.

## Supplementary Information

**Additional file 1: Figure S1.** Nucleotide sequences were analyzed in both directions. The two strains were considered to be co-infected if they were found to have reached more than half of the largest peak at the same site in the waveform, and if no noise was found in the surrounding waveform. If it was difficult to determine, a DNA insertion plasmid was used to analyze the sequence. **Table S1.** Number of *Plasmodium* cases in Binh Phuoc Province in 2003, 2012, 2015, and 2018.

## Data Availability

The data supporting the conclusions of this article are included in the article.

## Abbreviations

ACT: Artemisinin-based combination therapy
*Pfk13*: *Plasmodium falciparum* Kelch-13
RDT: Rapid diagnosis test
