# Supplementary material for: Reduction in Plasmodium falciparum Pfk13 and pfg377 allele diversity through time in southern Vietnam
Source: Trop Med Health. 2022 Mar 1;50:19. doi: 10.1186/s41182-022-00409-4 (PMC8887123; doi:10.1186/s41182-022-00409-4)
Supplement: Supplementary file 1 — Additional file 1: Figure S1. Nucleotide sequences were analyzed in both directions. The two strains were considered to be co-infected if they were found to have reached more than half of the largest peak at the same site in the waveform, and if no noise was found in the surrounding waveform. If it was difficult to determine, a DNA insertion plasmid was used to analyze the sequence. Table S1. Number of Plasmodium cases in Binh Phuoc Province in 2003, 2012, 2015, and 2018. [file 41182_2022_409_MOESM1_ESM.docx]

Additional file 1


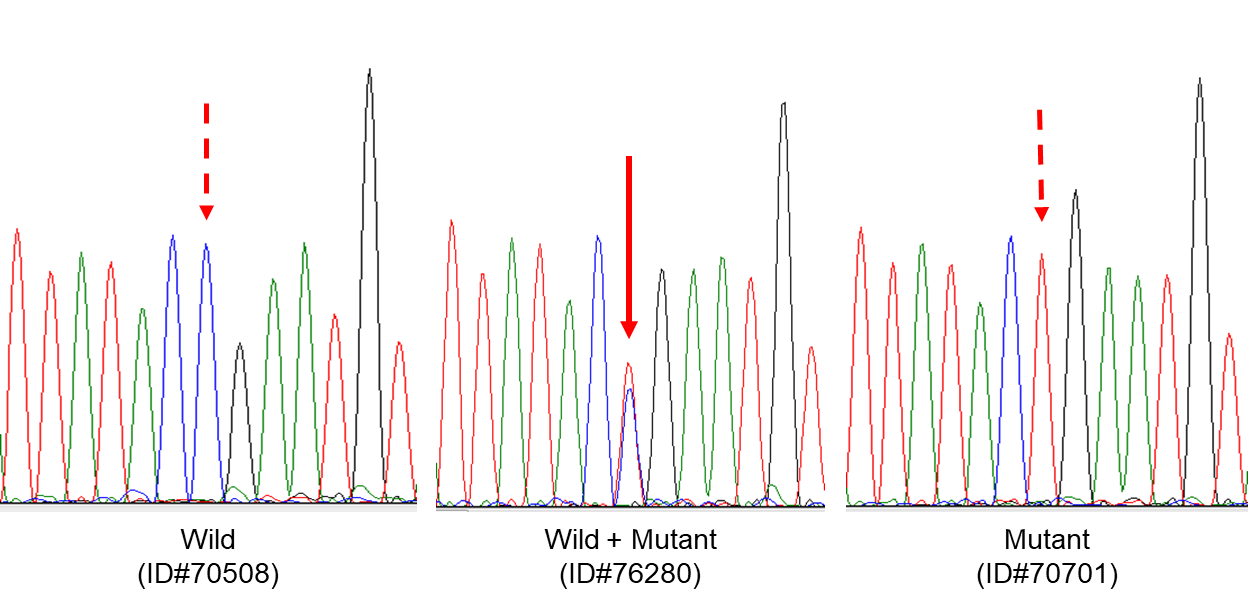


Fig. S1. Nucleotide sequences were analyzed in both directions. The two strains were considered to be co-infected if they were found to have reached more than half of the largest peak at the same site in the waveform, and if no noise was found in the surrounding waveform. If it was difficult to determine, a DNA insertion plasmid was used to analyze the sequence.

Table S1 Number of *Plasmodium* cases in Binh Phuoc Province in 2003, 2012, 2015, and 2018.

| Year | Total | Pf | Pv | Pf + Pv |  |
| --- | --- | --- | --- | --- | --- |
| 2003 | 3,953 | 3,263 | 676 | 14 |  |
| 2012 | 2,780 | 1,457 | 1,146 | 177 |  |
| 2015 | 1,799 | 1,000 | 706 | 93 |  |
| 2018 | 1,243 | 728 | 460 | 55 |  |
| Pf, *P. falciparum*; Pv, *P. vivax*; Pf + Pv, mixed infection with Pf and Pv | | | | |  |
|  |  |  |  |  |  |
